# A Syrian Refugee in Iraq Diagnosed as a Case of IL12RB1 Deficiency in Japan Using Dried Blood Spots

**DOI:** 10.3389/fimmu.2019.00058

**Published:** 2019-01-25

**Authors:** Lika'a Fasih Y. Al-Kzayer, Ahmed K. Yassin, Khalid Hama Salih, Tomonari Shigemura, Kenji Sano, Ruwaid Behnam Y. Al-Simaani, Miyuki Tanaka, Yozo Nakazawa, Yusuke Okuno

**Affiliations:** ^1^Department of Pediatrics, Shinshu University School of Medicine, Matsumoto, Japan; ^2^Department of Medicine, College of Medicine, Hawler Medical University, Erbil, Iraq; ^3^Department of Pediatrics, College of Medicine, Sulaymaniyah Medical University, Sulaymaniyah, Iraq; ^4^Department of Pathology, Iida Municipal Hospital, Iida, Japan; ^5^Department of Pediatrics, Noorjan Medical Complex, Erbil, Iraq; ^6^Center for Advanced Medicine and Clinical Research, Nagoya University Hospital, Nagoya, Japan

**Keywords:** IL12RB1 deficiency, mendelian susceptibility to mycobacterial diseases (MSMD), Bacillus Calmette-Guérin (BCG), Flinders Technology Associates (FTA), tuberculosis (TB), non-tuberculous mycobacteria (NTM), whole exome sequencing (WES), Iraq

## Abstract

Mendelian susceptibility to mycobacterial diseases (MSMD) is a rare condition of primary immunodeficiency disorder. Interleukin-12 receptor β1 (IL12RB1) deficiency, is the most common genetic etiology of MSMD, which is characterized by the selective predisposition to clinical disease caused by weakly-virulent mycobacteria, such as Bacillus Calmette-Guérin (BCG) vaccines, and environmental non-tuberculous mycobacteria (NTM). To the best of our knowledge, this is the first case of *IL12RB1* deficiency to be reported from Iraq. Our case is an 8-year-old Syrian girl, for first-cousin parents, with a refugee-status in the North of Iraq. She had a history of disseminated BCG infection 2 months after receiving BCG vaccine, in addition to repeated episodes of mild or severe illnesses, such as maculopapular skin rash, lymphadenopathy, gastroenteritis, meningitis, and clinically diagnosed tuberculosis (TB) based on local TB-prevalence setting. Because of limited medical facilities in the war-torn countries; in Syria and Iraq, no diagnosis could be reached. We used Flinders Technology Associates (FTA) cards to transfer her bone marrow aspirate to Japan. A homozygous *IL12RB1* mutation was detected by whole exome sequencing in Japan, using genomic-DNA extracted from dried bone marrow sample spots on FTA filter paper. In conclusion, diagnosis of MSMD due to *IL12RB1* deficiency was possible by transferring the FTA sample of the patient for genetic evaluation in Japan. Our report recalls the need of pediatricians in countries with TB-prevalence and high parental consanguinity, to consider *IL12RB1* deficiency in the differential diagnosis of a child with clinical evidence of TB, especially with the history of disseminated BCG disease.

## Introduction

Primary immunodeficiency (PID) disorders are diseases that result from developmental or functional defects in the immune system. Mendelian susceptibility to mycobacterial disease (MSMD) is a rare condition among the growing list of PID disorders, caused by genetic defects in the mononuclear phagocyte/T-helper-cell type-1 pathway, and is characterized by the selective predisposition to clinical disease caused by weakly virulent mycobacteria, such as Bacillus Calmette-Guérin (BCG) vaccines, and environmental non-tuberculous mycobacteria (NTM), in otherwise healthy individuals (1, 2). Affected individuals are also vulnerable to the more virulent mycobacterial species, including; *Mycobacterium tuberculosis* (M. TB), systemic salmonellosis, either typhoidal or, more commonly, non-typhoidal type, as well as candidiasis, and more rarely to infections with other intramacrophagic bacteria, fungi, or parasites, and possibly, a few viruses (2–5).

The most common genetic etiology of MSMD is the autosomal recessive (AR), complete interleukin-12 receptor β1 (IL12RB1) deficiency (MIM #614891), caused by the bi-allelic mutations in the *IL12RB1* gene (2, 3). IL12RB1 is a common receptor chain of the IL-12 and the IL-23 receptors and deficiency of IL12RB1 causes a profound defect in both IL-12 and IL-23 signaling. Upon infection with intracellular bacteria, phagocytes are activated and produce various cytokines, including IL-12 and IL-23. However, natural killer (NK) and T-cells from patients with *IL12RB1* deficiency, do not respond to IL-12 and thus, impair the production of interferon (IFN)-γ (3, 6–9).

The advanced technology of whole exome sequencing (WES), provides coverage of more than 95% of the exons, which harbor the majority of the genetic variants associated with human disease phenotypes (10). Likewise, Flinders Technology Associates (FTA) cards have proved successful for dried blood spot archiving, transportation, DNA extraction, and genetic sequencing. The filter paper matrix of the FTA card is impregnated with chaotropic agent that denatures infectious agents, and thus the sample is no longer considered a biohazard. Because of the small size of the FTA cards, they are convenient for storage in a limited space and transport of specimens (11, 12).

The purpose of this paper was to report a case of *IL12RB1* deficiency in a Syrian girl living in a TB-endemic area in Iraq, who had a history of disseminated BCG-disease, and recurrent clinical evidence of TB, along with frequent infectious episodes, that were treated aggressively with antibiotics and anti-TB agents, without a clear diagnosis, until it became possible, by transferring her bone marrow aspirate (BMA) sample to Japan via FTA cards and performing WES. To the best of our knowledge this is the first case of *IL12RB1* deficiency to be reported from Iraq.

## Background

### Case Presentation

**Figure 1:**
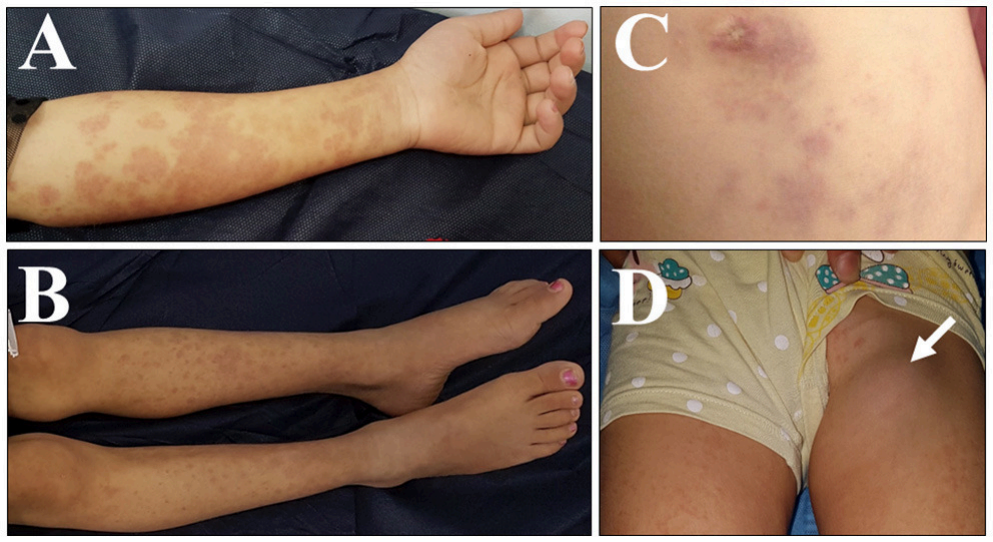
Clinical presentation of the case with *IL12RB1* deficiency. Pictures were taken on different occasions: **(A–D)** skin lesion in the form of multiple erythematous maculo-papular rash, over the upper limbs **(A)**, lower limbs **(B)**, and over the abdominal wall surrounding the umbilicus **(C)**. **(D)** A swelling is shown in the left groin, due to left inguinal lymphadenitis (arrowed).

An 8-year-old girl for first-cousin parents, she is the second child among four girls of a Syrian family having a refugee-status at a camp in Sulaymaniyah, northern Iraq, since 2014. Our patient was born uneventfully in August 2010 and received BCG vaccine, according to the schedule at 7th day of age. Two months later, she developed ipsilateral axillary lymphadenitis followed by generalized lymphadenopathy. Meanwhile, features of disseminated BCG infection, including fever, weight loss, disseminated maculopapular rash, and hepatosplenomegaly, were manifested, and managed by a prolonged course of anti-TB medicines including isoniazid, and rifampin. According to the history taken from the mother, our patient had repeated episodes of non-specific illnesses, in form of relapsing/remitting maculopapular skin rash, oral thrush, respiratory infection, gastroenteritis, and urinary tract infections that were treated in an outpatient setting, in addition to one episode of meningitis treated at a hospital in Syria. At 4-year-old, as the family fled the war in Syria to a camp in northern Iraq, the child's condition was severely deteriorated and she became seriously ill with fever, night sweating, diarrhea, and poor appetite. Thus, she was referred to the intensive care unit at Hiwa Hospital in Sulaymaniyah, the northern province in Iraq. Upon admission she was toxic, cachexic, and feverish, with generalized lymphadenopathy including cervical, axillary, inguinal and epitrochlear lymph nodes. The lymph nodes were multiple, asymmetrical, and visibly enlarged with the biggest about (3.5 × 3 cm) at left axilla, firm in consistency, not tender, and discrete. The abdomen was distended with the presence of hepatosplenomegaly and ascites, in addition to right lung crepitation. The patients' growth parameters were below the third centile. Investigations showed an erythrocyte sedimentation rate (ESR) of 110 (normal range 3–13) millimeters/hour (mm/h), along with hypochromic microcytic anemia, leukocytosis, and high immunoglobulin-G assay. Ascetic fluid showed lymphocytic predominance with a serum-to-ascites albumin gradient of < 1.1 gm/dl, normal liver, and renal function tests. HIV and hepatitis screening were negative. Chest X-ray and computed tomography (CT) of the chest and abdomen showed a pulmonary consolidation at the right lower lung, in addition to mesenteric lymphadenitis disclosed by CT. Although microbiological and histopathological evaluations were not done, there was a high index of suspicion of mycobacterial infection, either in the form of relapsing disseminated BCG disease or active TB, based on the TB-prevalent situation at the area of the camp. Furthermore, the patient did not respond to an initial course of broad-spectrum antibiotics. Thus, she was treated empirically with 4 anti-TB medications for 12 months, including; isoniazid, rifampin, pyrazinamide, and streptomycin that was later changed to ethambutol. She showed a very good clinical and laboratory responses. Several months later, after stopping anti-TB therapy, she relapsed with generalized lymphadenopathy and maculopapular skin rash (Figure 1).

She also had episodes of abdominal pain and bloody diarrhea, disturbed sleep, and weight loss. Our patient underwent several excisional biopsies from axillary, cervical, and groin lymph nodes, in Syria and in Iraq, but the results were non-conclusive. Moreover, during the periods of suspected infection with leukocytosis and lymph node neutrophilic infiltration, culture was not regularly done, mostly because of the limited laboratory facilities and being treated in an out-patient setting. There was no history of BCG disease or TB, among family members. On most occasions the patient had an ESR of ≥ 100 mm/h, hypochromic microcytic anemia, leukocytosis, neutrophilia, lymphopenia with hypercellular marrow examination, and low CD3 and CD4 by flowcytometry. Antinuclear antibody, in addition to toxoplasmosis, rubella, cytomegalovirus, herpes simplex, HIV, and syphilis, as well as the culture for TB, were all negative. Thyroid, liver, and renal function tests were normal.

### Sample Collection and Results

Upon consultation from Iraq, we used Whatman classic FTA cards (cat no. WB120205, GE Healthcare UK Limited, Buckinghamshire, UK), to transfer her BMA sample to Japan. The BMA drops were applied on the 4 circles of the classic FTA cards, using 125 μl per each circle. Two cards were consumed with a total of 8 circles were filled, dried for 1 h, kept in a special FTA envelope (cat no. WB100036, GE Healthcare) and shipped by plane at room temperature to Japan. DNA was extracted from the dried blood spots on the FTA cards as described previously (11, 12), and then was subjected to WES (13). The analysis covered >95% of the whole coding region and revealed a homozygous *IL12RB1*: (NM_005535) c.64+2T>G splice site mutation. The mutation was in a region of run of homozygosity, which covered the whole region of chromosome 19 p. Thus, our case had a complete *IL12RB1* deficiency.

Given that the living status of the family was unstable, hematopoietic stem cell transplantation was not a possible option. Therefore, prophylactic antibiotic was given, including azithromycin/trimethoprim-sulfamethoxazole (cotrimoxazole), along with the consideration of IFN-γ augmentation accordingly. Genetic counseling about the AR inheritance was provided to the family as well.

## Discussion

In this report, we described a case of MSMD due to the complete *IL12RB1* deficiency in a girl of a Syrian family, who survived the difficult war situation in Syria, and fled to a camp at northern Iraq. Since it was difficult to perform adequate medical evaluation under such complicated living situations in war-torn countries, her condition was regarded as an immune deficiency of unknown cause, until the etiology could be made clear in Japan. Our patient is a product of a consanguineous marriages of first-cousin parents. Of note, consanguineous marriages in the Middle-Eastern countries were reported to be as frequent as 50% or more, with Saudi Arabia, Iraq, Syria and Iran, being among the top of the list countries, followed by Turkey and Morocco (14). Accordingly, many AR-PID disorders such as complete *IL12RB1* deficiency, were first described in patients originating from these countries (1).

A mutation in the *IL12RB1* gene encoding the IL-12 receptor β chain is the most common genetic etiology of MSMD. *IL12RB1* deficiency is a rare disorder characterized by a predisposition to recurrent and/or severe disease caused by poor pathogenic mycobacteria and salmonellae. Host defense mechanism against mycobacteria including M. TB and NTM, as well as salmonella, depends principally upon the functional integrity of the IFN-γ/IL-12 pathway (3). Given that IFN-γ is a fundamental factor in the elimination of both TB and NTM, genetic defects in the IFN-γ pathway; including a mutation in the *IL12RB1* gene, result in MSMD. Accordingly, BCG diseases and salmonellosis are the most common infections documented in patient with *IL12RB1* deficiency, followed by Candida, NTM and TB (2). Of note, severe cases of TB were reported in patients with *IL12RB1* deficiency who live in highly TB-endemic areas of the world.

Our case had the typical presentation of disseminated BCG disease early in her life, which manifested the MSMD, with further repeated episodes of infection including a clinical evidence of TB. Without a clear microbiological evaluation, it was difficult to decide whether our case had the fluctuation course of disseminated BCG disease, or TB. However, both of disseminated BCG disease, and TB are treated similarly by anti-TB agents.

Indeed, TB represents a major health problem in developing countries such as Iraq and Syria. According to the World Health Organization (WHO), Iraq is among the countries of the Eastern Mediterranean region with a high prevalence of TB. TB incidence in Iraq was documented in 2014 to represent a rate of 43/100,000 per year with a case detection rate of 54%, and an incidence of 31/100,000 per year was reported in Sulaymaniyah in northern Iraq in 2010 (15). It was noted that patients with *IL12RB1* deficiency developed clinical TB in the absence of any personal or familial history of clinical disease by weakly virulent mycobacterial species (2, 16, 17). Therefore, TB infection in our case is not a remote possibility. Notably, it is essential to confirm the diagnosis of TB in suspected cases; however, this is still not widely possible in many underdeveloped countries where clinical diagnosis remains acceptable. The WHO report estimated that out of 5.2 million new and relapsed pulmonary TB cases in 2014, only 3 million (58%) were bacteriologically confirmed (15). The clinical presentation of a patient with *IL12RB1* deficiency, may vary in accordance with the ethnicity or country of residence, TB burden, BCG vaccination policy and the likelihood of exposure to virulent organisms. Our case had a homozygous *IL12RB1* c.64+2T>G, splice site mutation. Splice site mutation resulting in premature stop codons is one of the most common mutations of *IL12RB1* deficiency, in which a premature termination of translation in the extracellular domain occurs, leading to a complete *IL12RB1* deficiency (18).

FTA card was the most useful tool along with WES to solve the problem of this case. In our previously published work (Al-Kzayer et al), (11, 12, 19, 20), we used FTA-derived RNA and DNA to evaluate the genetic background of Iraqi children with acute leukemia, by performing different types of polymerase chain reaction as well as Sanger DNA sequencing. However, in this report we used the more advanced next-generation sequencing technology. WES using FTA-derived DNA was satisfactory in terms of sequencing coverage, sensitivity, and error rate. Advanced genetic diagnostic technologies are widely available in the developed world, whereas there is no access to such facilities in countries with limited-resources. Thus, collaboration in this aspect, with the use of simple tools to collect, store, and ship the dried blood spots to the country with the advanced setting, will remarkably help to achieve the diagnosis (21).

Studies that include series of PID cases from Iraq or Syria are needed to be genetically evaluated using WES, in order to estimate the frequency of MSMD diseases in such countries. Since AR-PID disorders are not uncommon in Middle-Eastern countries with the high consanguineous marriages, disclosure of the frequency of such inherited diseases with the understanding of its clinical presentation is helpful for the clinicians to expect the patients' diagnosis even in the absence of genetic analyses. Of note, the presence of consanguinity makes genetic diagnoses easier, as the pathogenic allele is most likely on the run of homozygosity regions. Thus, diagnostic yield may be higher in such countries compared with others.

In conclusion, our work demonstrates the importance of the international collaboration via FTA cards and its impact on the diagnosis of patients with genetic disorders in countries with limited-resources. Moreover, our report recalls the need of pediatricians in countries with TB-prevalence and high parental consanguinity, to consider IL12RB1 deficiency in the differential diagnosis of a child with clinical evidence of TB, especially with the history of disseminated BCG disease.

## Ethics Statement

In accordance with the Declaration of Helsinki, written informed consent was obtained from the parents of the patient for publication of this case report and the accompanying images, in addition to the ethical consideration of the confidentiality of the unrelated results disclosed via WES. The study was approved by Shinshu University School of Medicine, Institutional Review Board.

## Author Contributions

LA-K conceptualized and designed the study, and also wrote the paper. AY, KHS, MT, RA-S, and YN contributed to evaluating the clinical features and wrote the paper. TS and KS performed the immunopathological evaluation and wrote the paper. YO designed the study, performed the genetic analysis, and wrote the paper.

### Conflict of Interest Statement

The authors declare that the research was conducted in the absence of any commercial or financial relationships that could be construed as a potential conflict of interest.

## Abbreviations

AR: Autosomal recessive
BMA: Bone marrow aspirate
FTA: Flinders Technology Associates
MSMD: Mendelian susceptibility to mycobacterial diseases
NTM: Non-tuberculous mycobacteria
PID: Primary immunodeficiency disorders
WES: Whole exome sequencing.
